# Cathepsin B pulls the emergency brake on cellular necrosis

**DOI:** 10.1038/cddis.2016.76

**Published:** 2016-03-31

**Authors:** F S Wouters, G Bunt

**Affiliations:** 1 Laboratory for Molecular and Cellular Systems, Institute of Neuropathology, University Medical Center Göttingen, Göttingen, Germany; 2 Centre for Nanoscale Molecular Physiology of the Brain, Göttingen, Germany; 3 Clinical Optical Microscopy, Institute of Neuropathology, University Medical Center Göttingen, Göttingen, Germany

Cell death can take many shapes. Programmed ‘apoptotic' and uncontrolled ‘necrotic' cell death mark the extremes of the spectrum of possibilities.^1^ Whereas the induction of apoptosis follows distinct signaling steps and cleanly removes a cell from its tissue, necrosis represents the cell's wholesale disintegration and is in general harmful to the organism. Massive cellular insults lead to necrosis. It appears, however, that cells have some choice in the mode of their demise depending on the severity of the insult. Hypoxic cores in solid tumors^2^ or perfusion-impaired brain tissue in stroke,^3^ for instance are typically necrotic, but possess an apoptotic shell.

The same can be seen for lysosomal damage.^4, 5, 6^ Lysosomes can rupture during ischemic or traumatic cell injury,^7^ giving rise to both apoptotic and necrotic outcomes.

Lysosomes are cellular organelles with a proteolytic function and possess an impressive set of protease enzymes^8^ dedicated to the degradation of both intracellular material, such as damaged or old organelles, and extracellular components such as matrix proteins. The release of the lysosomal proteases into the cytosol sentences the cell to death by autodigestion.

In a recent *Cell Death Discovery* article,^9^ we looked at the fate of the major regulators of apoptosis, the bcl-2 protein family, in order to understand how cell fate upon lysosomal rupture is directed toward necrosis or apoptosis. This protein family contains both pro- and anti-apoptotic members and has been shown to be part of the proteolytic spectrum of cathepsin lysosomal proteases.^10^ We reasoned that a regulated degradation of these proteins, by affecting the equilibrium of pro- and anti-apoptotic signaling, might offer a means for switching between cell death forms.

To this end, we imaged the proteolytic degradation of the major bcl-2 family representatives bcl-xl, bid and bax upon induced lysosomal disruption in real time using förster resonance energy transfer (FRET) microscopy of single cells. We created proteolytic FRET sensors by sandwiching full-length bcl-2 proteins between cyan and yellow fluorescent proteins. A strong FRET signal is detected in the intact sensor, which is lost upon cleavage (Figure 1, left).

The bcl-2 sensors show a common sigmoidal ‘delay-snap' cleavage behavior: after a delay of tens of minutes, cleavage occurs rapidly and is completed within minutes. Among the observed bcl-2 members, anti-apoptotic bcl-xl is degraded before pro-apoptotic bid and bax. Cells thus appear to accumulate pro-apoptotic reactants in the early stages of lysosomal rupture.

The pro-apoptotic bid showed a conspicuous behavior. Before the overall sigmoidal degradation, a pronounced and very rapid cleavage was observed in the first minutes of lysosomal lysis. Fast proteolytic processing of bid is used in apoptosis and might serve the same role in lysosomal lysis. In apoptosis, the caspase 8 protease activates bid by its truncation, directly connecting the extrinsic and intrinsic apoptotic signaling pathways. This mechanism is called the ‘bid shunt' and processing of bid by cathepsin B has been suggested to operate similarly.^11^ Our work places the bid shunt very early in lysosomal cell death signaling and this is the first time this event is caught ‘on film'.

In order to gain a better understanding of the regulation of bcl-2 protein levels by cathepsins and the involvement in programmatic steps in necrosis and apoptosis, we investigated the interplay of both systems in more detail.

Given the relatively low amount of lysosomes that were disrupted in the early stages after lysosomal disruption, we selected the neutral-active cathepsin B as likely candidate for most of the early proteolytic actions. Hence, we repeated the experiments in the presence of a selective cathepsin B inhibitor and an inhibitor for the thiol-cathepsin class, to which cathepsin B belongs. Inhibition of the thiol-cathepsins confirmed their predominant role in the degradation of bcl-2 family proteins, as the proteolysis of all three bcl-2 constructs was inhibited. Selective cathepsin B inhibition helped uncover its role in steering cell death. We found that early bid truncation was abolished, confirming cathepsin B as the responsible protease. Furthermore, when we limited lysosomal protease action to the time window of the early bid truncation, we detected late-apoptotic caspase 3/7 activation, showing that cells had chosen an apoptotic exit.

Unexpectedly and paradoxically, bax and also bid exhibited a new accelerated proteolytic behavior upon selective inhibition of cathepsin B. The delay phase before the onset of cleavage was completely abolished, in particular for bax. As a general inhibition of the thiol-cathepsin classes resulted in the complete inhibition of the proteolysis of these same sensors, we concluded that their accelerated degradation upon cathepsin B inhibition is most likely caused by another, unidentified, thiol-cathepsin. This proteolytic cascade prevents the degradation of apoptosis-supporting proteins, shifting the balance toward apotosis. The identity of this cathepsin requires further research.

Cells thus appear to be able to pull an ‘emergency brake' upon lethal injury with damaging of lysosomes. Starting with cathepsin B, an early programmatic protease cascade is initiated that appears to avoid necrosis in favor of apoptosis. In this apoptotic exit effort, cathepsin B preferentially degrades anti-apoptotic bcl-xl, activates bid by its rapid and selective truncation, and degrades a thiol-cathepsin to avoid the removal of apoptotic bid and bax (Figure 1, right). Up to a certain level of lysosomal damage, this three-pronged rescue program steers dying cells away from necrosis. When massive damage overwhelms the cell with thiol-cathepsins that eat away at essential cellular proteins, necrosis becomes unavoidable. Our research contributes to the understanding of cell death on a molecular mechanistic level by uncovering programmatic steps at the interface between necrosis and apoptosis. Knowledge on cell death decision signaling is therapeutically relevant, for example, in the formulation of more effective chemotherapy and in combatting toxic drug side effects.

## Figures and Tables

**Figure 1:**
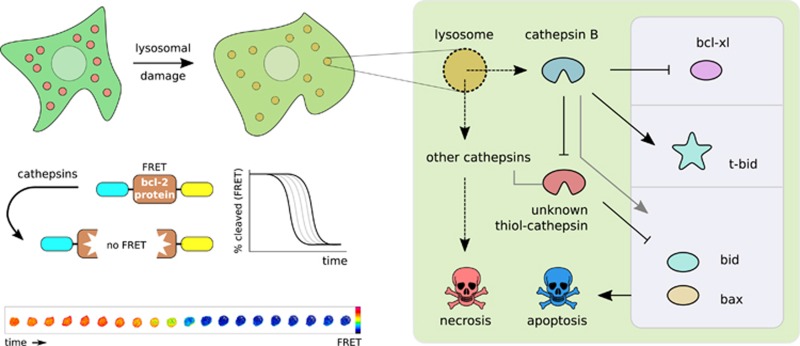
Left: upon lysosomal damage, cathepsin proteases are released in the cytosol, finally resulting in cell death. The apoptosis-regulating bcl-2 family proteins bcl-xl, bid and bax are cleaved by cathepsins. Their degradation was followed by FRET microscopy on single cells in real time. Cleavage of FRET sensors consisting of full-length bcl-2 proteins sandwiched between cyan and yellow fluorescent protein was imaged. The intact sensors show FRET, the cleaved sensors do not. Right: cathepsin B initiates an apoptotic exit program by (i) proteolytic removal and consequent inactivation of anti-apoptotic bcl-xl, (ii) the rapid and controlled proteolytic activation (t-bid) of pro-apoptotic bid, and (iii) the proteolytic inactivation of an unknown thiol-cathepsin that would otherwise have removed pro-apoptotic bax and bid. Even in the background of a strong necrotic stimulus as lysosomal lysis, cathepsin B thus appears to launch an early apoptotic exit program. FRET, förster resonance energy transfer; t-bid, truncated bid.
